# Accurate characterization of complex Bloch modes in optical chain waveguides using real-valued computations

**DOI:** 10.1038/s41598-023-48477-8

**Published:** 2023-12-13

**Authors:** Maryam Ghahremani, Mahmoud Shahabadi

**Affiliations:** Photonics Research Laboratory, Center of Excellence on Applied Electromagnetic Systems, School of Electrical and Computer Engineering, College of Engineering, University of Tehran, Tehran, Iran

**Keywords:** Applied optics, Integrated optics

## Abstract

This research presents a highly accurate and easy-to-implement method to characterize the complex Bloch modes propagating along optical chain waveguides with three-dimensional (3D) layered geometries and dispersive negative-epsilon material compositions. The technique combines commercial EM solver results with analytical post-processing to avoid iterative complex root estimation on the complex plane. The proposed methodology is based on the real-valued computations that yield the complex Bloch wavevector with superior accuracy even when both radiation and material losses are present. In addition, we introduce a single unit-cell technique to provide the possibility of dense meshing of 3D geometries when available computational resources are limited. To verify our results, two different plasmonic and dielectric case studies are discussed. The obtained results agree well with numerical and experimental results from the literature. Due to its generality, robustness, and high accuracy, the method is beneficial for studying a large variety of waveguide-based nanophotonic components.

## Introduction

Periodic optical waveguides like their microwave counterparts consist of an array of identical elements separated by a certain distance along the wave propagation direction^1^. Over recent years, different realizations of these waveguides have been presented in both two-dimensional (2D) and three-dimensional (3D) geometries. The well-known examples are coupled-resonator optical waveguides (CROWs)^2,3^, nanoparticle chain waveguides^4,5^, and subwavelength gratings (SWGs)^6,7^. They have shown several advantages in geometry and guided-mode properties over uniform (non-periodic) waveguides. For instance, they offer the possibility of implementing circuits with sharp bends^8,9^ and designing filters with almost any desirable frequency responses^10^. This renders them useful for numerous applications in integrated nanophotonic devices such as optical delay lines^11^, passive filters^10,12^, couplers^13^, and polarizers^14^, to name a few. To profit from periodic waveguides in the aforementioned devices, one needs to know the propagation characteristics of their guided Bloch modes. As an example, in the Bragg deflector couplers, the imaginary part of the Bloch wavevector can be used to accurately estimate the scattering strength of the structure which is an essential parameter for the design process. Therefore, developing a rigorous and efficient method for characterizing the Bloch modes in a layered waveguide subjected to open boundary conditions is of prime importance in the analysis and design of several optical devices. Due to its importance, this subject has been investigated by many researchers from the past years for simple 2D structures^15^ to the present day for complicated 3D structures^4,16,17^. In what follows, we first provide an extensive review of the existing methods.

### Semi-analytical methods

#### 2D geometries

For a 2D optical waveguide with one-dimensional (1D) periodicity, a commonly used approach to characterize the Bloch eigenmodes is the Fourier modal method (FMM). A representative FMM technique is rigorous coupled-wave analysis (RCWA)^18^ where the permittivity function of the periodic layer and EM fields are expanded in a Fourier series and Floquet-Fourier series, respectively. This leads to a matrix equation for the unknown expansion coefficients. Applying RCWA to the modal analysis of a 2D array waveguide requires determining the complex roots of a nonlinear system^19^. Several techniques have been proposed to find these complex roots. One approach is a root-searching technique described in Ref.^20,21^, which uses an iterative algorithm like Newton’s method to find the unknown complex roots. To accurately compute the roots, this method depends on an initial point sufficiently close to the actual roots. It also requires matrix truncations with large values^20,21^ to ensure convergence. A non-iterative method called the Cauchy Integration Method has also been presented^21^. This finds complex roots in a predetermined closed region of interest but requires extra computational cost from contour integration over the region’s boundary. Another approach is the full-wave Source-Model Technique (SMT)^15,22^ applicable to both lossless and lossy waveguides. The frequency-domain SMT uses an integral equation formulation with a spectral representation of the periodic Green’s function for an array of elementary sources. It enables modal analysis of periodic arrays of arbitrary smooth cylinders in free space^15^, or in a triple-layered geometry^23^. Other similar methods are the generalized multipole technique (GMT)^24^ and the lattice sums (LS) technique^25^ for periodic waveguides. As with the above methods, the characteristic equations from these techniques must be numerically solved for their complex roots.

#### 3D geometries

Depending on the structure of the periodic waveguide, several semi-analytical methods exist to calculate complex-valued Bloch wavenumbers. The coupled-dipole approximation (CDA) is a common semi-analytical method for modal analysis of a linear chain of nanoparticles (NPs) in a uniform host^26^. This approach is valid for sufficiently small particles, or frequency ranges near the dipolar resonance, where the EM fields of the lowest-order spherical multipoles (dipoles) dominate. A generalized dipole approximation method has recently been developed to identify modal properties along arbitrary-shaped NP chains in a homogeneous medium^16^. However, considering all inter-particle interactions for lossy arrays requires analytic continuation to the domain of complex wavenumbers and frequencies. For a NP chain in a planar layered medium, an eigen-decomposition (ED) analysis has been presented^27^. The ED method solves for scattering spectra over real-valued frequencies and wavenumbers, avoiding tedious root searching on the complex plane. The frequency bandwidth has been interpreted to estimate the quality factor of guided modes; however, calculating the imaginary part of the complex wavenumber is not possible. For coupled-resonator optical waveguides (CROWs), the guided modes can be approximated using an optical analogue of the tight-binding (TB) model from condensed-matter physics^2^, or by means of transfer matrices^28^. The TB model is suitable for approximate modal analysis of structures where non-nearest-neighbor cavity couplings are negligible. For subwavelength grating (SWG) waveguides, the dispersion diagram can be estimated by first calculating the effective index of the waveguide. This requires exploiting approximate calculations from effective medium theory or the effective index method^29^. To calculate the imaginary part of the effective refractive indices, a 3D FMM analysis based on 2D RCWA has been recently developed^30^.

### Numerical methods

Apart from some exceptions, the aforementioned semi-analytical techniques primarily deal with periodic waveguides with simple geometries. For more complex geometries, modal dispersion and attenuation can only be determined numerically using full-wave EM solvers. The major challenge for numerical full-wave methods is modeling an infinitely long periodic structure to fully account for all inter-particle interactions. To address this, several techniques have been introduced so far. A common technique is the numerical eigensolver approach^31^ which is capable of solving for the guided modes of periodic structures with complex geometry and arbitrary material composition. The eigen-solver module in COMSOL Multiphysics utilizes this approach. With this solver, periodic structures are simulated along a single period and the obtained complex eigenfrequencies enable calculation of both the dispersion diagram and quality factor of the modes. The same modal information can also be generated using the finite-difference time-domain (FDTD) method, where the total power spectral density recorded by point-like monitors provides resonant peaks representing the establishment of an optical mode^32^. In both the eigensolver and FDTD approaches, the spatial decay, which is related to the imaginary part of the complex wavenumber, has to be characterized indirectly^31,33^. One strategy for this is to link the modal losses to the spectral bandwidth of the excited eigenmode through the group velocity^31^. Only for very low levels of modal losses does this calculation lead to reliable loss estimations^34^. Another strategy is based on full-wave simulation of a finite number of unit cells with a source to view the mode profile and obtain the propagation length^33^. However, the accuracy of this straightforward approach is limited by computational resources. Very recently, an iterative FEM-based technique in the frequency domain has been presented^17^ that analyzes 3D geometries in a homogeneous environment but requires iterative searching on the complex plane.

This review surveys existing methods to calculate complex wavenumbers for guided modes in periodic optical waveguides. Many techniques estimate complex roots of a nonlinear characteristic equation by searching on a complex plane. The estimated attenuation constant is often inaccurate due to applied approximations. According to the authors, precise calculation of wavelength-dependent propagation loss has been limited, especially for 3D complex geometries.

Rather than estimating the complex roots of a characteristic equation, we derive a real-valued equation that governs the optical intensity on the midplane of a Fabry–Perot (FP) resonator. The resonator is formed by terminating the periodic waveguide under study with two perfect mirrors. Since the derived equation contains the phase and attenuation constant of the guided modes, we obtain modal constants with high accuracy by fitting this equation to the calculated optical intensity. To reduce computational costs, we present a method that analyzes a single unit cell of the periodic structure. This general technique can be implemented with any electromagnetic solver that supports periodic boundary conditions, including finite element method (FEM) or finite-difference time-domain (FDTD) solvers. Our method can provide highly accurate estimates of modal attenuation constants, even when attenuation arises from both radiative and lossy regions within the periodic structure. No iterative search is required and all the electromagnetic interaction effects are considered. The method is widely applicable for analyzing a broad range of optical periodic structures. To demonstrate the superior accuracy and robustness of our proposed method, we compare our results to Bloch modes previously characterized using other techniques, including the rigorous full-wave SMT analysis^23^, and FDTD band structure technique^32^.

**Figure 1:**
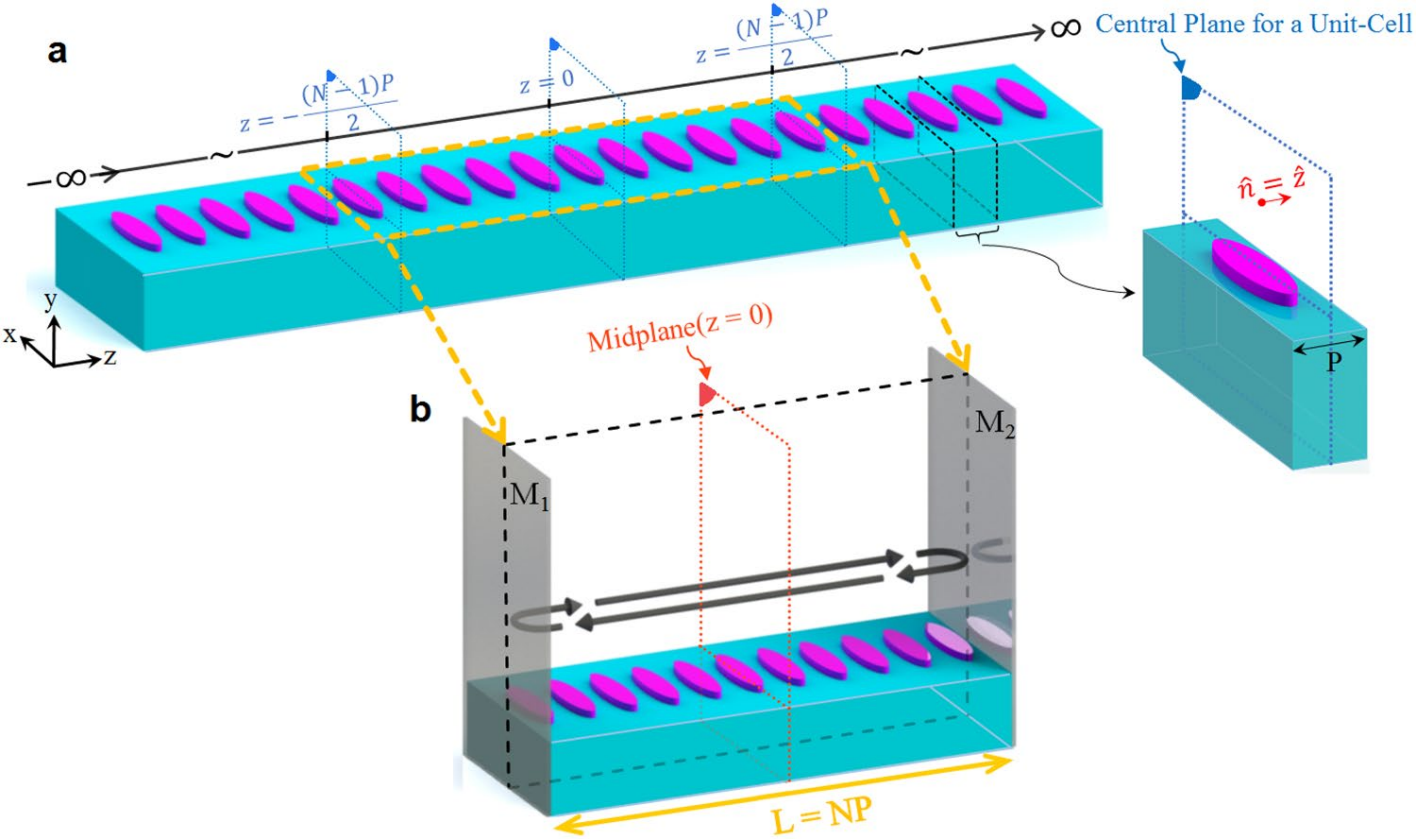
(**a**) An infinitely long one-dimensionally periodic optical waveguide consisting of dispersive unit-cells being separated by a period of *P* along the wave propagation direction (the *z*-axis); (**b**) the optical Fabry-Perot resonator of length $L = NP$ which is constructed by inserting perfect mirrors M1 and M2 at symmetry planes of $z = - {{NP} / 2}$ and $z = + {{NP} / 2}$. Here, we assume $N = 11$.

## Discussion

### Bloch-mode characterization

Consider the general case of an infinitely long one-dimensionally (linear) periodic waveguide consisting of unit-cells with arbitrary material composition being separated by a period of *P* along the wave propagation direction (the *z*-axis), as shown in Fig. 1a. Note that the structure is unbounded along *x* and *y* axes. For a given real frequency $\omega$, our proposed method aims to characterize the proper modes of the waveguide structure in the presence of radiation or material losses. These proper modes are the source-free EM fields that can propagate along the periodicity axis with a constant Bloch wavenumber. This wavenumber is considered in the lower half of the complex plane when the time-harmonic function is assumed to be of the form exp $\left( +j \omega t\right)$, as is the case throughout the paper. As the modes of interest are proper, the lateral wavevector components are required to satisfy the radiation (outgoing-wave) condition. A formal mathematical statement of the outgoing-wave condition for periodic structures is described in Ref.^35^.

#### Proposed methodology avoiding complex root calculation

In this subsection, we propose our methodology to obtain the complex Bloch wavenumber of the propagating modes in periodic waveguides. This technique is based on a real-valued mathematical relation allowing straightforward computation of the phase and attenuation constants of guided Bloch modes. It is valid both for uniform and periodic optical waveguides with any particle geometries symmetrical about the central plane of the unit cell (see Fig. 1a). The materials composing the waveguide and/or background medium can be dispersive, i.e., with frequency-dependent behavior, thus dissipative. To derive the required mathematical relation, we first terminate the waveguide under investigation by two infinitely large perfect mirrors whose normals are parallel to the propagation direction such that an optical FP resonator is formed by them as illustrated in Fig. 1b. When the origin of the coordinate system is at the center of the resulting FP resonator, these perfect mirrors are placed at $z = \pm {{{z_p}} / 2}$ . In the case of a periodic structure (Fig. 1), we have ${z_p} = {{NP}/2}$ where *N* is a positive integer number; hence, the length of the resonator is equal to the length of N unit cells of the waveguide. In general, this resonator possesses several complex natural frequencies. Their corresponding modes are eigensolutions of time-harmonic source-free Maxwell’s equations. The complex natural frequencies of the resonator govern the frequency response of any transfer function defined within the resonator. In other words, the resonance line shapes observed for any transfer function contain modal information of the guided eigenmodes propagating along the optical waveguide under investigation. With this in mind, we may excite the FP resonator, for instance, with an infinitesimal electric dipole represented by a volume current density whose phasor is given by $\vec {J}\left( {\vec {r}} \right) = I\vec {dl} {\delta _3}\left( {\vec {r} - {{\vec {r}}_{scr}}} \right)$ in which $I\vec {dl}$ and ${\vec {r} - {{\vec {r}}_{scr}}}$ denote the dipole moment and the location of this source, respectively. When magnetic excitation is considered, the infinitesimal dipole of magnetic current is similarly given by $\vec M\left( {\vec {r}} \right) = {I_{mag}}\vec {dl}{\delta _3}\left( {\vec {r} - {{\vec {r}}_{scr}}} \right)$ in which $I_{mag}\vec {dl}$ represents the magnetic dipole moment. Either of these two choices is commonly in favor of the numerical technique utilized for this problem. In this work, the selected volume current density is assumed to be located at the midplane of the resulting FP resonator configuration. Regarding the direction of the current element $\vec {dl}$, it should be oriented to match the polarization of the modes intended for the characterization. To define the transfer function, we assume that the output is the phasor of the electric field at a sample observation point on the midplane of the cavity, i.e., at ${\vec {r}_{obs}} = \left( {{x_o},{y_o},{z_o} = 0} \right)$ . Although in general the choice of the excitation and observation locations on the plane $z=0$ is not fundamentally important (this has been numerically tested by choosing multiple observation points in each case study), regions with expected high field values are favorable. As mentioned above, the total electric field in the resonator can be expanded as a linear combination of the resonator modes, i.e., as $\vec {E}\left( {\vec {r};\omega } \right) = \sum \limits _l {{A_l}\left( \omega \right) } {\vec {E}_l}\left( {\vec {r};\omega } \right)$ where the complex coefficient ${A_l}\left( \omega \right)$ is the amplitude of the *l*-th resonator mode whose electric field is represented by the phasor ${\vec {E}_l}\left( {\vec {r};\omega } \right)$^36^. As the source frequency approaches the resonance frequency of a resonator mode, i.e., when $\omega \approx {\omega _l}$, where ${\omega _l}$ stands for the real resonance frequency of the *l*-th resonator mode, most of the source energy is coupled to this mode and the coefficient ${A_l}\left( \omega \right)$ becomes the dominant amplitude, as a result of which the total field will be dominated by the field of a single resonator mode ${\vec {E}_l}\left( {\vec {r};\omega } \right)$.

Obviously, this resonator mode is composed of the forward and backward traveling waves of the *m*-th eigenmode of the periodic optical waveguide inserted between the two mirrors. Considering the above fact, we can derive the optical intensity or the intensity of the electric field for the *m*-th eigenmode, i.e., ${I_m}\left( \omega \right) = {\vec {E}_m}\left( {{{\vec {r}}_{obs}},\omega } \right) \cdot \vec {E}_m^*\left( {{{\vec {r}}_{obs}},\omega } \right)$ on the midplane of the resonator, i.e., at the point ${\vec {r}_{obs}} = \left( {{x_o},{y_o},{z_o} = 0} \right)$. This leads to the real-valued relation

1 $$\begin{aligned} {I_m}\left( \omega \right) = \frac{{{C_m}{e^{ + {\alpha _m}\left( \omega \right) L}}}}{{\cosh \left( {{\alpha _m}\left( \omega \right) L} \right) - \cos \left( {{\beta _m}\left( \omega \right) L} \right) }} \end{aligned}$$

which is valid for $\omega \approx {\omega _l}$. In this relation, $\alpha _m \left( \omega \right)$ and $\beta _m \left( \omega \right)$ are the attenuation and phase constant of the *m*-th eigenmode of the periodic optical waveguide under investigation, respectively, $C_m$ is a constant real coefficient, and *L* denotes the resonator length. In deriving Eq. (1), we have assumed an electric current density on the resonator midplane; consequently, perfect magnetic mirrors are placed on both ends of the FP resonator. Further details of the above derivation are given in “Methods”. Equation (1) enables us to characterize modal properties of an optical waveguide from the sole knowledge of the electric field intensity on the midplane of a FP resonator as a function of frequency $\omega$. After careful examination of Eq. (1), we realize that the maxima of this real-valued function occur at the real value $\omega$ for which

2 $$\begin{aligned} {\beta _k}(\omega ) = \frac{{k\,\pi }}{L} \end{aligned}$$

where *k* is an even integer number. The value of *k* is determined from the number of extrema of the established standing-wave pattern between the two mirrors. The phase constant of the *m*-th eigenmode of the periodic optical waveguide can thus be calculated using Eq. (2). Note that the spatial mode profiles are simultaneously monitored to distinguish different guided modes. For a given resonator length *L*, this approach determines the phase constant at specific frequencies. By analyzing the cavity for various lengths, we can arrive at additional points on the dispersion diagram and improve its frequency resolution. Having determined the modal dispersion characteristics, we are now able to calculate other modal properties independently and through the frequency lineshape of the excited resonance modes. To this end, we expand the phase constant $\beta _{m}\left( \omega \right)$ and the attenuation constant $\alpha _{m}\left( \omega \right)$ in the vicinity of the resonance frequency of the FP resonator (that is $\omega _l$ ) in terms of a truncated Taylor series as

3 $$\begin{aligned} {\beta _m}(\omega ) \approx {\beta _m}({\omega _l}) + (\omega - {\omega _l}){\left. {\frac{{d{\beta _m}(\omega )}}{{d\omega }}} \right| _{\omega = {\omega _l}}} \end{aligned}$$

4 $$\begin{aligned} {\alpha _m}(\omega ) \approx {\alpha _m}({\omega _l}) + (\omega - {\omega _l}){\left. {\frac{{d{\alpha _m}(\omega )}}{{d\omega }}} \right| _{\omega = {\omega _l}}} \end{aligned}$$

These expansions are substituted in Eq. (1). Thus, the optical intensity in the vicinity of the resonance peak at $\omega = \omega _l$ is approximated by

5 $$\begin{aligned} {I_m}\left( \omega \right) \approx \frac{{{C_m}{e^{\left( {{\alpha _m}\left( {{\omega _l}} \right) + \left( {\omega - {\omega _l}} \right) {{\left. {\frac{{d{\alpha _m}}}{{d\omega }}} \right| }_{\omega = {\omega _l}}}} \right) L}}}}{{\cosh \left( {\left( {{\alpha _m}\left( {{\omega _l}} \right) + \left( {\omega - {\omega _l}} \right) {{\left. {\frac{{d{\alpha _m}}}{{d\omega }}} \right| }_{\omega = {\omega _l}}}} \right) L} \right) - \cos \left( {\left( {{\beta _m}\left( {{\omega _l}} \right) + \frac{{\omega - {\omega _l}}}{{{v_g}\left( {{\omega _l}} \right) }}} \right) L} \right) }} \end{aligned}$$

where ${v_g}\left( \omega \right) = {{d\omega }/ {d{\beta _m}}}$ represents group velocity of the propagating eigenmode. The remaining modal parameters will thus be determined by fitting Eq. (5) to the computational data obtained using any full-wave EM solver. The fitting procedure is explained in “Methods” section.

Any guided modes excitable through the applied current source can be characterized by the proposed technique. For the ideal case of a completely lossless structure with no radiation leakage or material dissipation, i.e., $\alpha _m \left( \omega \right) = 0$, resonance modes exhibit extremely narrow linewidth. Identifying such modes necessitates precise wavelength scanning in the computationally generated spectral response. The purely evanescent modes, i.e., $\beta _m \left( \omega \right) = 0$, located within the stop band region represent cut-off waveguide modes. These modes can couple to the applied current source; however, they do not form standing waves along the resonator. As a result, they cannot be characterized by using Eq. (1).

As mentioned previously, the focus of this work is to obtain the complex propagation constant of the Bloch eigenmodes propagating along periodic optical waveguides. From the computational point of view, however, to numerically analyze a 3D resonator involving *N* unit cells is challenging as the large structure meshing influences the accuracy of the final results and the required computational resources. This becomes more crucial in the case of waveguides composed of plasmonic materials or complex geometries where a higher number of mesh cells is required. To avoid the possible cost of dense meshing and high computational resources, in what follows, we propose an alternative approach to restrict the computation to a single unit cell.

#### Synthetic realization of an FP resonator using a finite number of unit-cell analyses

This section presents a numerical technique that enables us to replace the analysis of an FP resonator made of *N* unit cells (Fig. 1b) with the analysis of a single unit cell *N* times. There are computational advantages to this approach. Even though we have to perform a number of analyses that match the number of unit cells in the original FP cavity, the computational effort remains strictly proportional to the number of unit cells. However, the computation time required for a single unit cell is much smaller than this time for the full model containing *N* unit cells. Furthermore, our proposed method realizes infinitely large end mirrors whereas a direct computation of an FP resonator with *N* unit-cells models finite-size end mirrors. Hence, this synthetic but exact realization of an FP resonator is the main advantage of the analysis to be introduced in what follows.

To describe the method of analysis, we consider an arbitrary symmetrical unit cell, such as the one depicted in Fig. 1a. The central unit-cell whose central plane coincides with the midplane of the original FP resonator $\left( z = 0 \right)$ is excited using a volume current density given by $\vec {J_{u}}\left( \vec {r} \right)$ and defined within a unit cell, i.e., for $\left| z \right| < {P / 2}$. The current density $\vec {J_{u}}\left( \vec {r} \right)$ is assumed to be zero otherwise. Now, for the realization of an FP resonator of length $L = NP$, the periodic boundary condition is enforced at the planes $z = \pm {P/2}$ where the phase difference between the two planes is assumed to be $\Delta \phi _n = 2n\pi /N$. For every integer value *n*, by enforcing this periodic boundary condition, one realizes a current distribution according to the relation

6 $$\begin{aligned} \vec {J}_{n}\left( {x,y,z} \right) = \sum \limits _{q = - \infty }^{ + \infty } {{{\vec {J}}_u}\left( {x,y,z - qP} \right) \exp ( - jq\Delta {\varphi _n})} \end{aligned}$$

The EM field generated by $\vec {J_n}\left( x,y,z\right)$ is stored at the observation points across the unit cell. This computation is carried out *N* times for $n = 0,1, \ldots ,N - 1$. Note that the normalized superposition of the current distributions given by (6) will be

7 $$\begin{aligned} \vec {J}_{total}(x,y,z) = \frac{1}{N}\sum \limits _{n = 0}^{N - 1} {{{\vec {J}}_n}(x,y,z)} = {\vec {J}_u}(x,y,z) \end{aligned}$$

for $\left| z \right| < {P / 2}$ and will be zero elsewhere in the FP resonator. In general, it can readily be shown that ${\vec {J}_{total}}\left( {x,y,z} \right) = {\vec {J}_u}\left( {x,y,z - n'P} \right)$ where $n'$ assumes the integer values $\left\{ {0,\mathrm{{ }} \pm N,\mathrm{{ }} \pm 2N,\mathrm{{ }} \ldots } \right\}$. The total current density $\vec {J}_{total} \left( x,y,z\right)$ is zero elsewhere. Therefore, the only nonzero current sources will be inside the unit cells whose central planes are at $z = 0,\mathrm{{ }} \pm NP,\mathrm{{ }} \pm 2NP$, etc. In other words, the infinitely long periodic waveguide is excited with an array of sources whose period is a multiple integer *N* of the waveguide period. According to the image theory, this is equivalent to the excitation of an FP resonator of length *NP* at its midplane with perfect mirrors located on $z = \pm NP/2$. It should be noted that the mirrors have infinitely large areas and can be forced to be either perfect electric conductors (PEC) or perfect magnetic conductors (PMC) depending on the properties of the driving current density $\vec {J}_u\left( \vec {r} \right)$ existing in the central unit cell.

The above analysis also provides the spatial distribution of the EM fields within the synthetically realized FP resonator. Let ${\vec {E}_n}\left( {x,y,z} \right)$ be the phasor of the electric field generated by $\vec {J}_n \left( x,y,z \right)$ and stored for a given *n* at specific observation points across the FP resonator, that is for $|z|<NP/2$. According to the principle of superposition, when the waveguide is made of linear time-invariant materials, we can determine the response to $\vec {J}_{total}\left( x,y,z \right)$ using the relation

8 $$\begin{aligned} {\vec {E}_{total}}(x,y,z) = \frac{1}{N}\sum \limits _{n = 0}^{N - 1} {{{\vec {E}}_n}(x,y,z)} \end{aligned}$$

In summary, by applying the aforementioned formulations to the numerical results obtained from N single unit-cell analyses, we can obtain the EM field inside the synthetically realized FP resonator terminated with infinitely large perfect end mirrors. From Eq. (1), we only require the total electric field on the midplane of the FP resonator whence both the phase and attenuation constant of the waveguide Bloch eigenmodes can be calculated.

**Figure 2:**
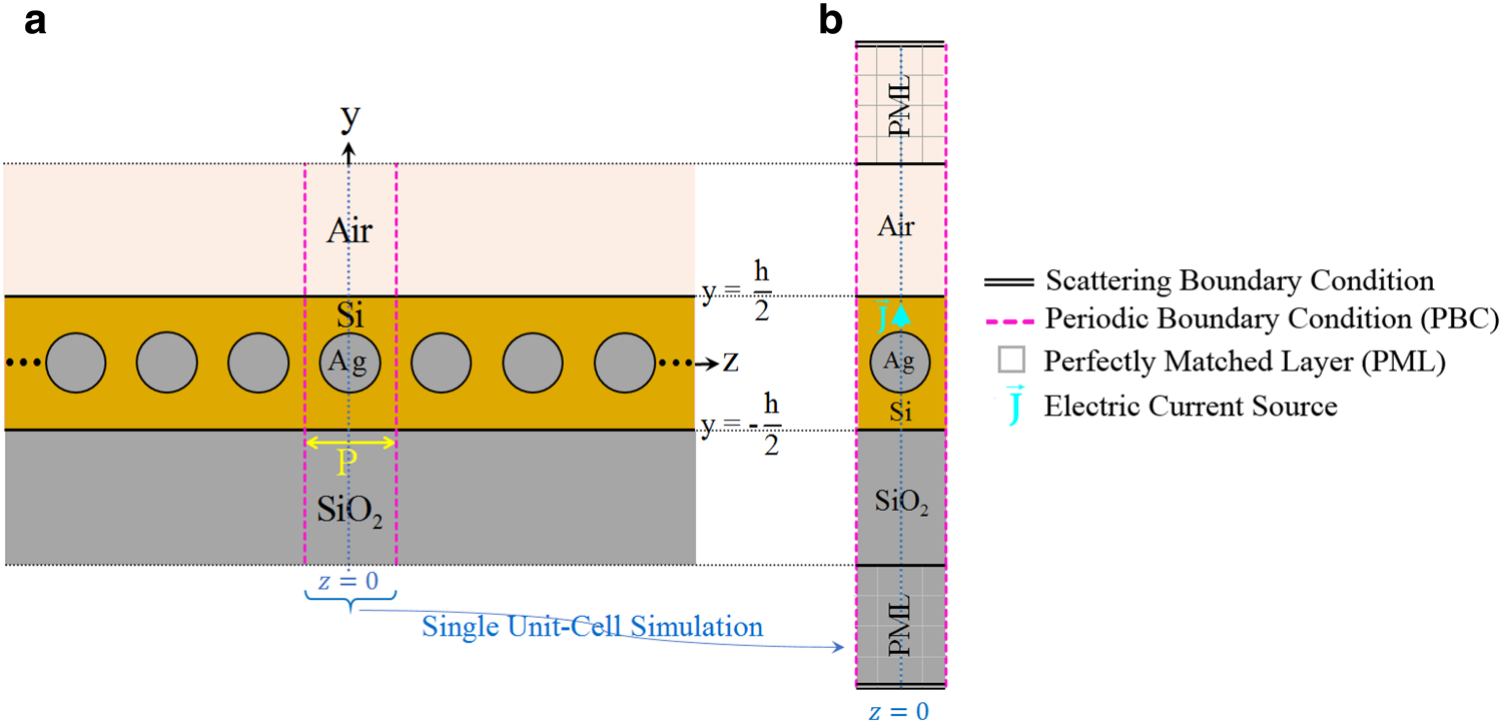
(**a**) A periodical array of plasmonic nanorods with a period of *P* resided in a silicon slab with a thickness of $h = 10R$. The Si slab is deposited on a glass substrate with a refractive index of $n_{SiO_2} = 1.51$ at $y = -5R$ and is covered by air. The structure is adopted from Ref.^23^. (**b**) Schematic diagram representing full-wave simulation configuration of a unit cell of the periodic waveguide which illustrates location of the enforced driving source. Configuration of the required FP cavity is realized by synthetically analyzing this single unit cell.

## Numerical results

To verify our numerical technique, in this section, two different plasmonic and dielectric structures are analyzed to determine their Bloch modes. The obtained results are compared to the Bloch modes previously calculated by the rigorous full-wave frequency-domain methods. For both examples, the cavity configuration is realized using the single unit cell analysis explained in the previous section. To obtain the cavity frequency response (transfer function), the structure under investigation is excited using an infinitesimal electric or magnetic dipole depending on the mode profile to be characterized. This excitation current is located on the midplane $\left( z=0\right)$ of the FP cavity. The generated EM field is then monitored at a well-chosen sample point on the cavity midplane to probe the cavity frequency response as a function of source frequency. The point probe detects the field intensity at the probe position. Through analyzing the obtained frequency response, the modal properties of various propagating Bloch modes of the periodic waveguide can be characterized at discrete frequencies. Not only does our method of analysis avoid tedious searching of the roots in a complex-number plane, a method that has been widely used in the existing literature, but also provides the value of propagation loss with superior accuracy.

All of the following full-wave simulations are performed in the platform of COMSOL MultiphysicsTM by utilizing four cores of a 64-bit PC (Intel quad-core CPU with 2.83 GHz clock speed and 24 GB RAM). The perfectly matched layer (PML) absorbing boundaries^37^ are incorporated into the cavity structure to emulate open boundary conditions and to truncate the computational domain. The PML layers are positioned $\lambda /2n_s$ away from the substrate and superstrate where $\lambda$ is the longest wavelength in the specified range and $n_s$ denotes refractive index of the layer. The excitation sources are chosen as an infinitely long out-of-plane line source for 2D and as a point dipole for 3D chain waveguides. The periodic Floquet boundary condition is applied on the unit cell planes perpendicular to propagation to account for inter-cell effects. To achieve the synthetic realization of the FP resonator with the length of $L = NP$, a single unit cell is simulated *N* times with the Floquet k-vector defined as $k_n = 2n\pi /NP$ with $n \in 0,\ldots , N-1$ providing the required phase difference among excitation sources. The fields from these unit cell simulations are extracted and post-processed using the equations discussed in the previous section to determine properties of the corresponding FP resonator.

### Linear chain of plasmonic nanowires embedded in a triple-layered structure

As an example of a possible periodic configuration of interest within the frame of the present work, the case of a 2D plasmonic optical waveguide (invariant in the out-of-plane direction, i.e., the *x*-axis) is considered. The structure is composed of a linear chain of metallic nanowires (NWs) embedded in a triple-layered structure, as illustrated in Fig. 2a. In this example, we compare the results obtained using our method with those provided by the available rigorous analysis. The rigorous full-wave frequency-domain SMT method has been chosen to make such a comparison^23^. The prime reason motivating us to discuss this example is to confirm that our proposed approach can generate highly accurate results for optical plasmonic array waveguides which are more complicated in terms of analysis than all-dielectric counterparts.

**Figure 3:**
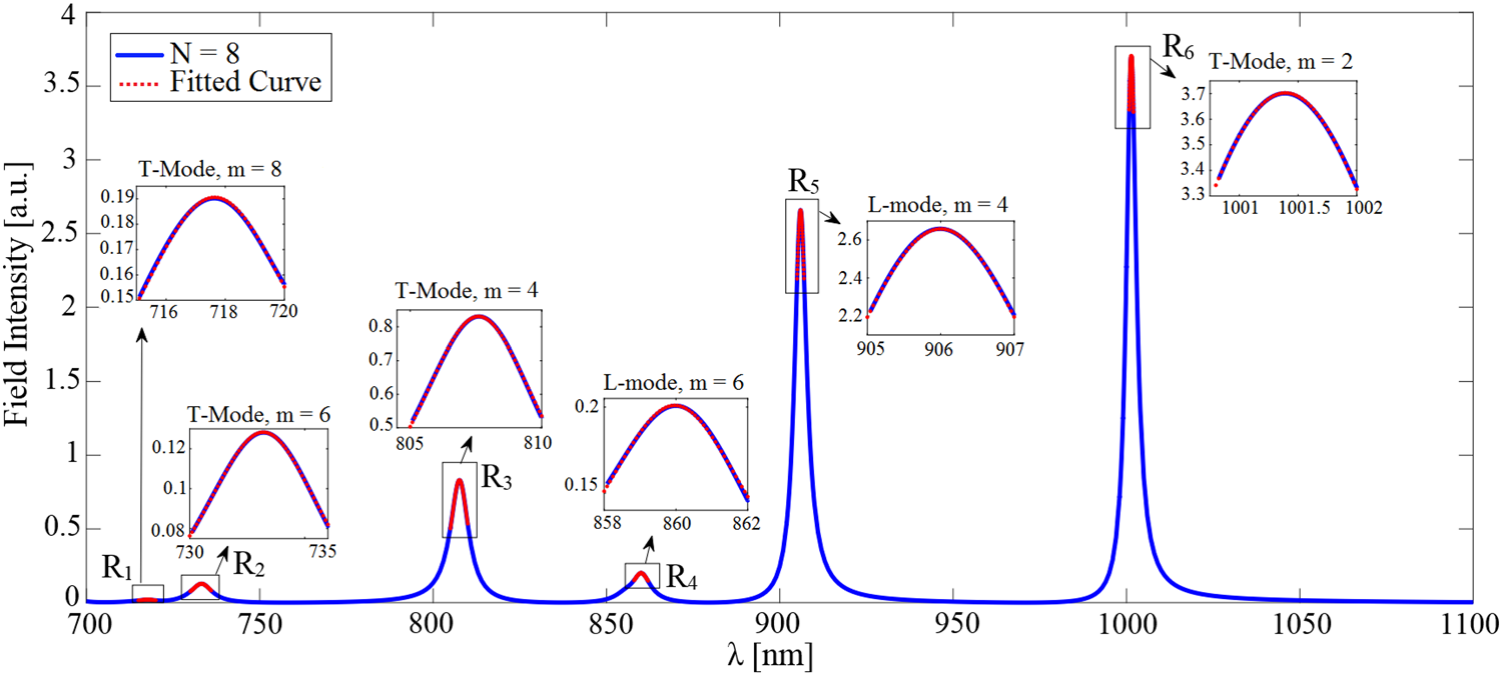
Simulated electric field intensity spectrum of the first two TM-polarized propagating modes which is monitored at a sample observation point $\left( x_o, y_o, z=0 \right)$ for the FP cavity of length $L = 8P$ $\left( N = 8 \right)$ with the choice of $R = 20$ nm and $P = 50$ nm. The required FP cavity configuration is realized using synthetic analyses of a single unit cell whose central plane coincides with the plane of $z = 0$, as explained in the previous sections. In terms of the driving source parameters, an infinitesimal electric dipole is employed inside the Si slab which flows parallel to the *x*-axis and is located on the central plane of the unit cell. A high-resolution simulation has been carried out close to the resonance frequencies to locally trace the exact spectral line shapes of the excited resonance modes. The red dotted lines represent fitting of our proposed model (i.e., Eq. (5)) to the intensity spectrum near the resonance frequencies.

**Figure 4:**
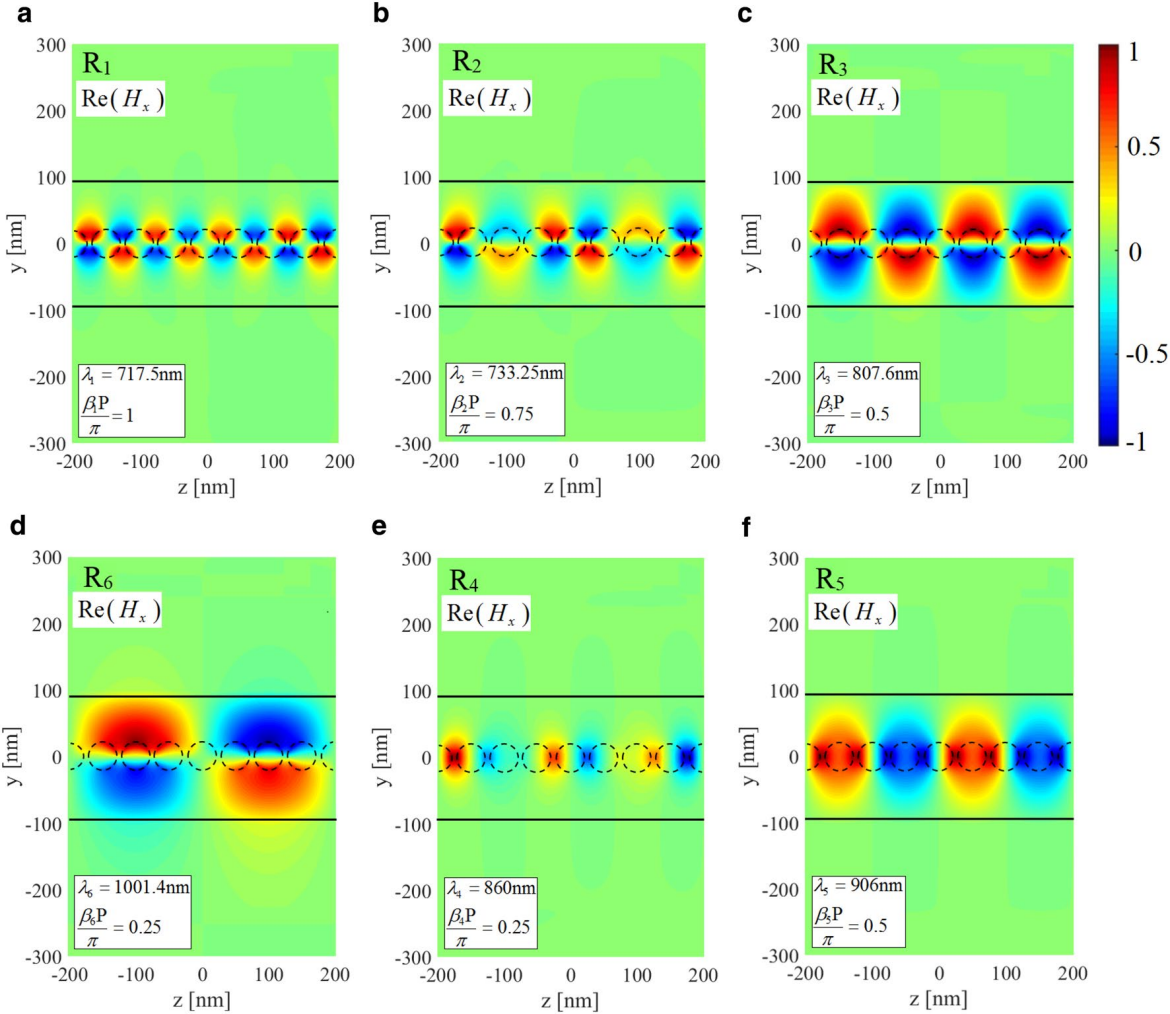
Normalized *x*-component of the magnetic field at resonance frequencies observed in the spectrum of Fig. 3: (**a–d**) T-mode, (**e**) and (**f**) L-mode. Using the obtained field profiles, the longitudinal mode number is determined to calculate the phase constant corresponding to each resonance frequency according to Eq. (2).

The NWs are assumed to be made of silver whose equivalent complex permittivity follows empirical data reported by Johnson and Christy^38^. The radius of NWs is chosen as $R= 20$ nm, and the period of the chain is $P = 50$ nm. The refractive index data for the silicon slab is adopted from^39^, and its thickness is taken as $h = 10$ R. The silicon layer is deposited on a half-space glass substrate with $n_{glass} = 1.52$ at $y = -5$ R while the upper layer is air (see Fig. 2). The properties of the excitation current should be deliberately chosen to match the spatial field profile of the resonance modes intended for study. Here, we are seeking the propagation properties associated with the first two TM-polarized guided modes of the structure. Thus, the structure is excited using an infinitesimal electric dipole represented by $\vec {J}$ which is located on the central plane of the unit cell and oriented parallel to the *y*-axis inside the Si slab, as illustrated in Fig. 2b. Due to the exciting current pattern, a non-zero distribution of the electric field on the midplane of the synthesized FP resonator is ensured. Only resonance modes with spatial field distribution accurately congruent with the imposed excitation current will be strongly excited and made observable. Modes mismatched to the current in their field configurations may not be appreciably excited. As both the L-mode and T-mode exhibit *y*-directed electric field amplitude centered on the midplane of the FP resonator, this current source will effectively drive both modes. The constructed FP configuration corresponds to Fig. 9a discussed in “Methods”. The normalized source frequency $\omega L/2\pi c$ is swept over the spectral range of interest for a given value of *N*. The complex *y*-component of the electric field phasor, i.e., ${E_y}\left( {{{\vec {r}}_{obs}},\omega } \right)$ with ${\vec {r}_{obs}} = ({x_o},{y_o},z = 0)$, is computed where the observation point $\vec {r}_{obs}$ is located inside the Si slab. Figure 3 represents the spectrum of the electric field intensity, i.e., $I\left( \omega \right) = {E_y}\left( {{{\vec {r}}_{obs}},\omega } \right) E_y^*\left( {{{\vec {r}}_{obs}},\omega } \right)$, for $N = 8$. To characterize the Bloch-mode phase constant associated with each of the observed resonance frequencies, we have determined the EM field pattern of the established standing wave on the longitudinal section of the resonator. For this purpose, the *x*-component of the magnetic field at the observed resonances is computed; the results are depicted in Fig. 4. The obtained field profiles belong to the two first TM-polarized modes of the waveguide, i.e., longitudinal mode (L-mode) and transverse mode (T-mode), which are to be characterized in this section. The spectral response at shorter wavelengths is required to identify higher-order TM modes.

**Figure 5:**
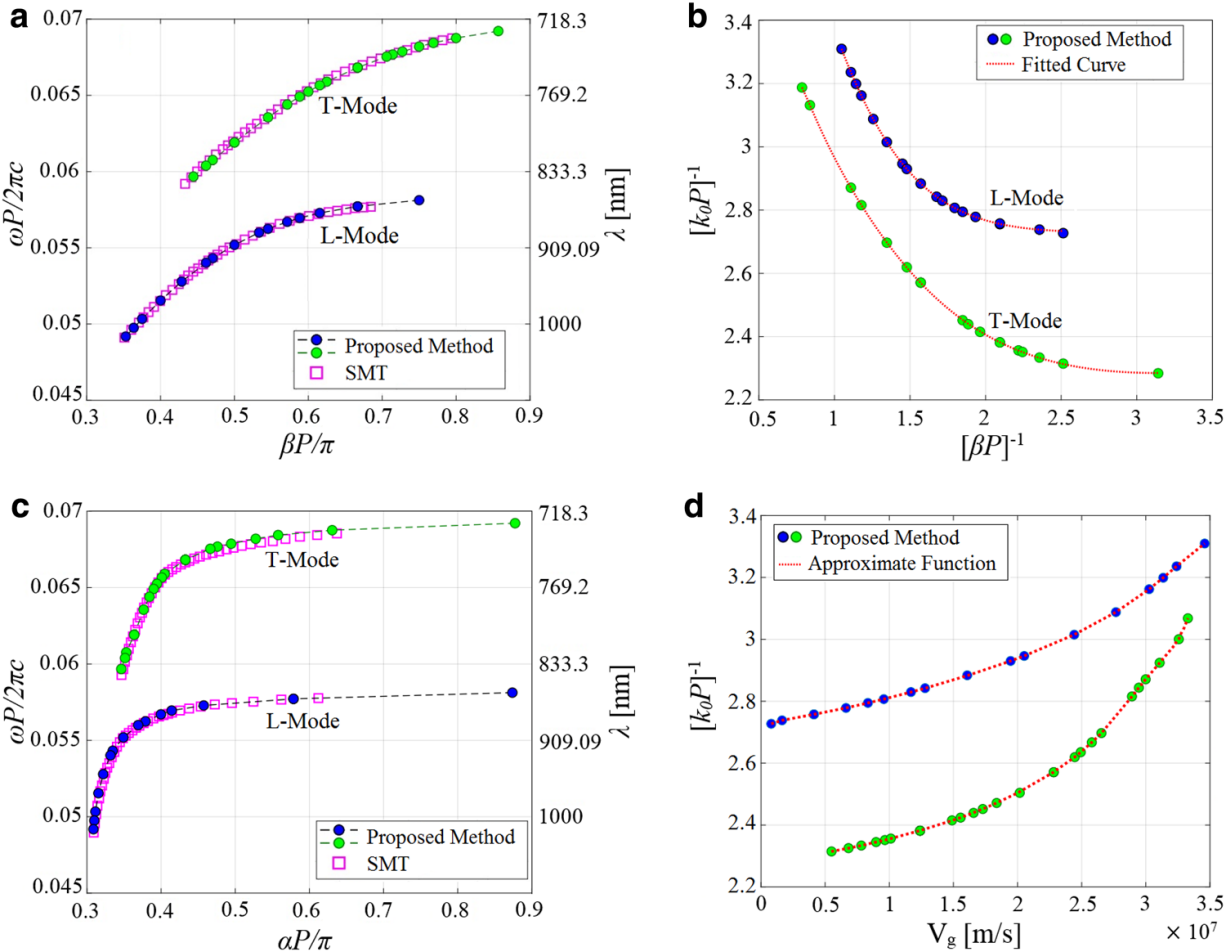
(**a**) Calculated phase constants of the first two TM-polarized guided modes (L and T modes, respectively) of the structure under consideration (Fig. 2) which are obtained through analyzing the synthesized FP resonator for different lengths (blue and green circles) and are compared with those provided by rigorous SMT technique^23^ (purple blocks). (**b**) Fitting our approximate model of the dispersion relation presented by Eq. (9) to the phase constants calculated by our proposed method. (**c**) attenuation constant, and (**d**) group velocity calculated for the afore-said parameters.

Repeating similar procedure for different values of *N* (different lengths of the FP resonator), we have arrived at the dispersion diagrams for both of the L- and T-modes. The obtained numerical results are shown in Fig. 5a. They agree very well with those provided by the SMT technique^23^. Using the determined dispersion diagram along with the simulated resonance linewidth, we calculate the remaining modal parameters including the Bloch-mode attenuation constant and group velocity in what follows. According to “Methods”, the optimal initial guess values of the group velocity at resonance points are first found by approximating Eq. (26) as

9 $$\begin{aligned} \begin{array}{c} \frac{1}{{{k_0}P}} = f\left( {\beta P} \right) \approx {c_0} + \frac{{{c_1}}}{{\beta P}} + \frac{{{c_2}}}{{{{\left( {\beta P} \right) }^2}}} + \frac{{{c_3}}}{{{{\left( {\beta P} \right) }^3}}} + \frac{{{c_4}}}{{{{\left( {\beta P} \right) }^4}}} + \frac{{{c_5}}}{{{{\left( {\beta P} \right) }^5}}} \end{array} \end{aligned}$$

where the unknown constant coefficients are calculated by fitting this model to the actual dispersion data obtained for both modes. It can be seen that the fitted curves (the red dotted lines shown in Fig. 5b) match closely to those numerically determined so that the constants for the L-mode are obtained as $c_1 = 3.91$, $c_2 = 16.99$, $c_3 = 3.91$, $c_4 = -25.11$, $c_5 = 7.235$, and for T-mode are obtained as $c_0 = 3.43$, $c_1 = -9.05$, $c_2 = 24.85$, $c_3 = -29.056$, $c_4 = 16.48$, $c_5 = -3.69$. Therefore, the optimal initial guess value for the group velocity at each resonance frequency is provided by Eq. (9). The Bloch-mode attenuation constant and group velocity can thus be straightforwardly extracted by fitting the transfer function given by Eq. (5) to the computed intensity spectra in the vicinity of the resonances. The fitted curves are depicted as the red dotted lines in Fig. 3.

Having accomplished a similar analysis for different values of *N* (i.e., different resonator lengths), we have calculated the attenuation constant and group velocity versus frequency, as shown in Fig. 5c,d, respectively. Our obtained attenuation constants align with those calculated in Ref.^23^ (purple blocks) using the SMT technique.

**Figure 6:**
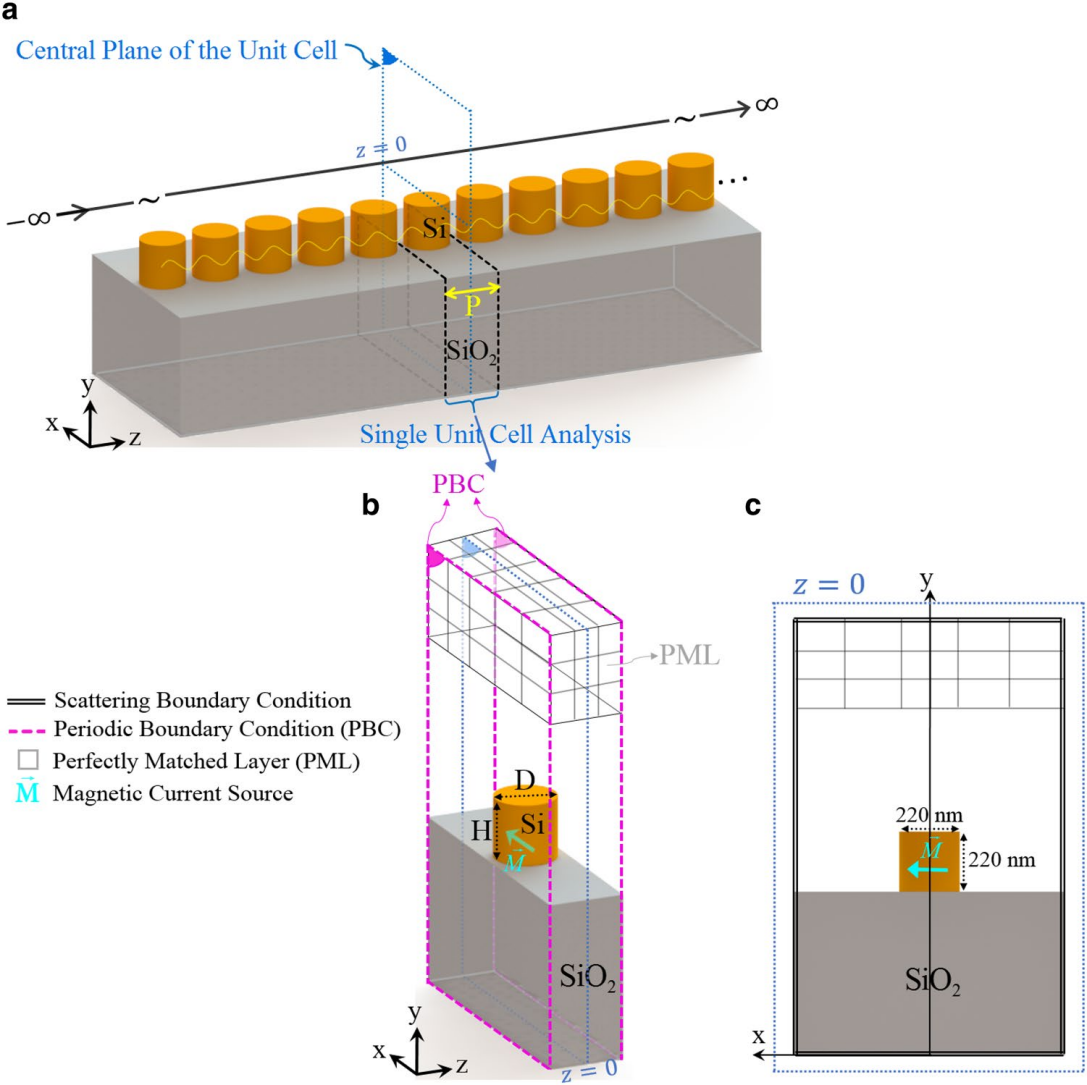
(**a**) Schematic view of the investigated array waveguide consisting of Si cylindrical nanoparticles with a diameter *D* and a height *H* of 220nm. The particles reside on top of a quartz substrate with a period of *P* along the wave propagation direction (*z*-axis). A gap of 50 nm exists between two neighboring nanoparticles. (**b**) Schematic diagram representing single unit cell simulation configuration in which location of the applied source is illustrated. The synthetic FP resonator is realized by numerical analyses of this single unit-cell (**c**) Schematic representation of $z = 0$ cross-section of the unit cell.

### Linear chain of high-index dielectric nanoparticles on a quartz substrate

In our second example, we study modal properties of a linear chain of high-index cylindrical dielectric nanoparticles located on a quartz substrate (Fig. 6) operating at near-infrared (NIR) wavelengths. Here, we are motivated to narrow the modal analysis of this example to a single unit cell simulation and generate highly accurate data using our proposed solution scheme as we confirmed in the previous example. To examine the numerical accuracy, our obtained results for the Bloch mode dispersion and attenuation are compared with those we have calculated using the FDTD band structure method^32^ and the measurement data provided by^33^, respectively.

**Figure 7:**
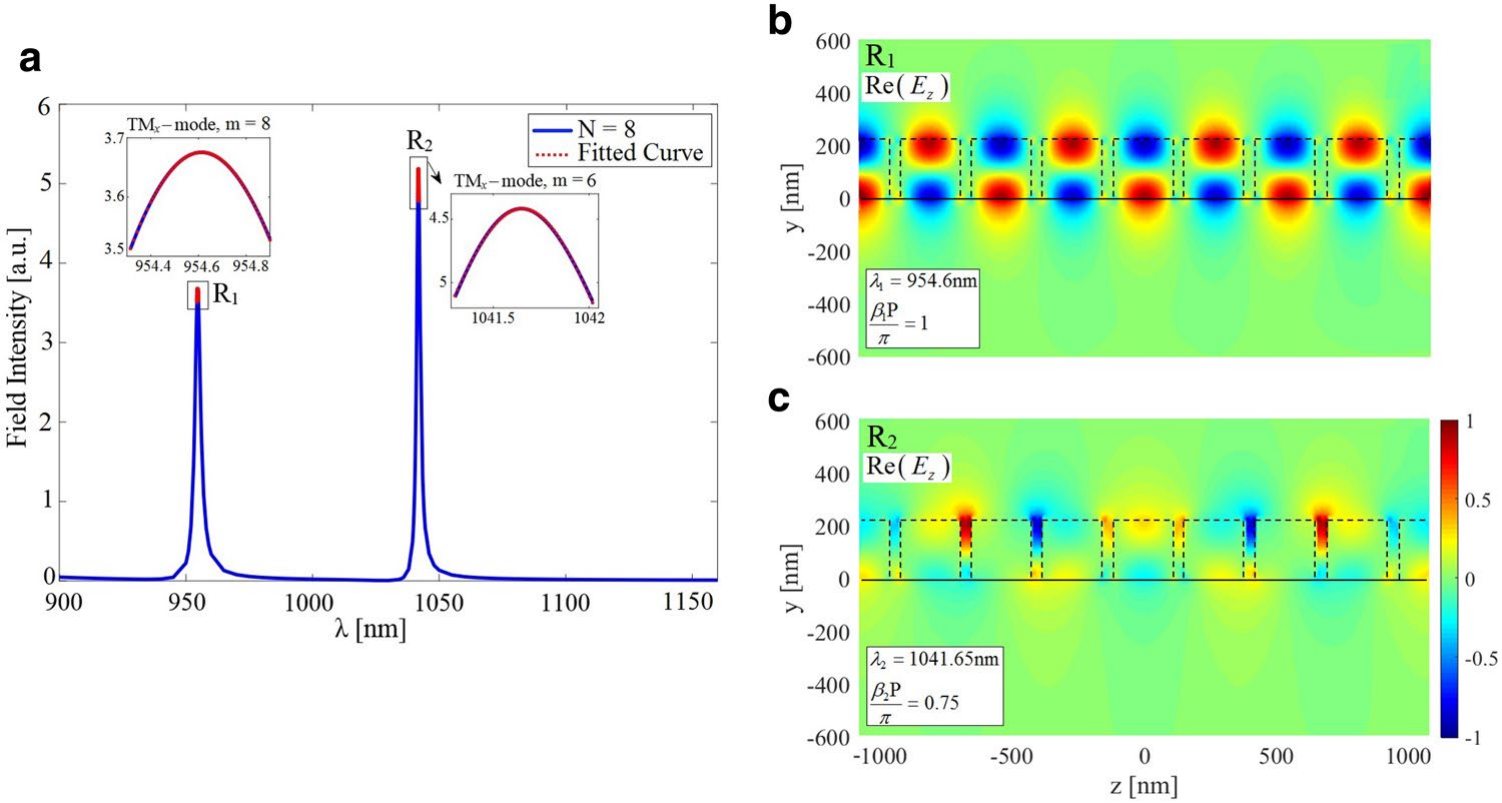
(**a**) Simulated intensity spectrum of the FP resonator of length $L = 8P \left( N = 8 \right)$ constructed from the periodic waveguide shown in Fig. 6a. This spectrum is monitored at an observation point on the resonator midplane $\left( x_o, y_o, z=0 \right)$ with the parameters of $D = H = 220$ nm and $g = 50$ nm. The FP resonator is synthesized using the single unit cell analysis. As for the structure excitation, an infinitesimal *x*-directed magnetic dipole is applied inside the Si nanoparticle. The red dotted lines represent fitting of our proposed model (i.e., Eq. (5)) to the intensity spectrum near resonance frequencies. Normalized *z*-component (longitudinal) of the electric field for the TMx-polarized guided mode at the two resonance frequencies $R_1$ (**b**) and $R_2$ (**c**) observed in the frequency response.

**Figure 8:**
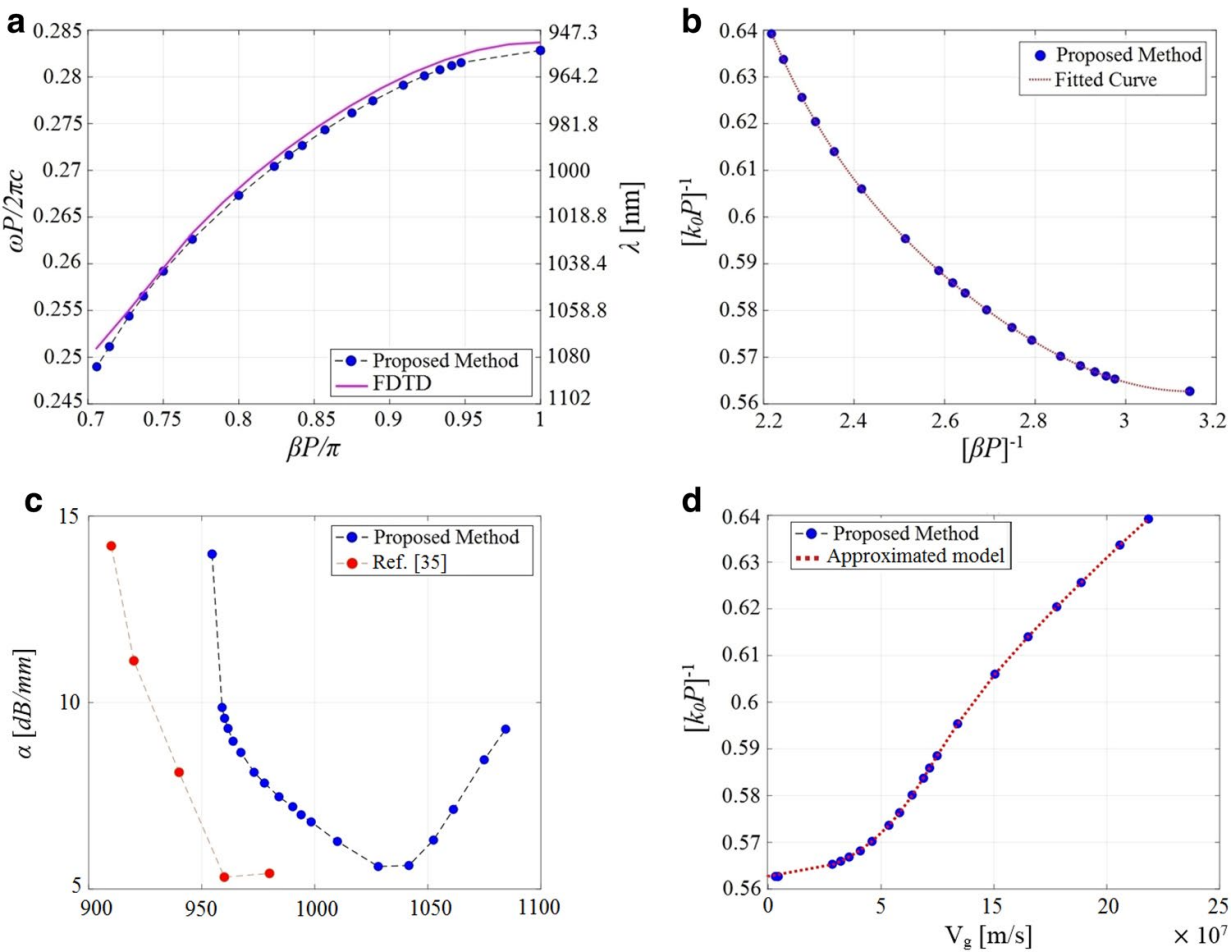
(**a**) Modal dispersion diagram of the fundamental TMx-polarized guided mode which propagates along the Si nanoparticle chain waveguide shown in Fig. 6. The numerical results obtained using our proposed method (blue circles) are compared with those we have calculated using the FDTD band structure technique (solid purple line). (**b**) Fitting our model of the dispersion relation (Eq. 9) to the phase constants achieved by our proposed method to compute the unknown coefficients. (**c**) Modal loss calculation of the fundamental TMx-polarized guided mode. Our numerical results (blue circles) compare well with those presented in Ref.^33^ (red circles) except for a wavelength shift. (**d**) Blue circles represent group velocities calculated using our proposed method which are thoroughly matched to the results obtained using Eq. (9) (dotted line).

**Figure 9:**
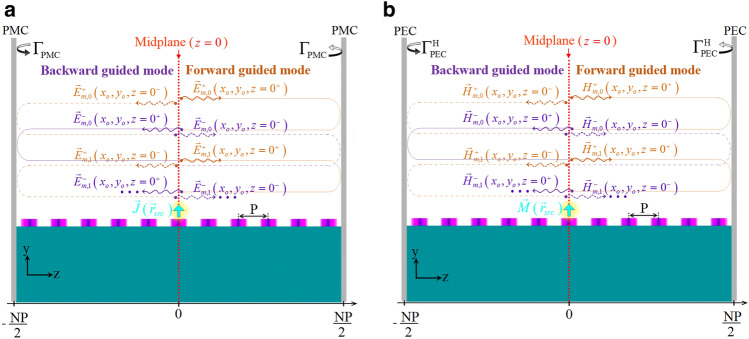
Longitudinal cross-section of the constructed FP resonator illustrated in Fig. 1b. (**a**) The structure is terminated by two infinitely large PMC planes and is excited by an electric current density on the resonator midplane, (**b**) The dual problem when the FP resonator is terminated by two infinitely large PEC planes and is excited by a magnetic current density on the resonator midplane.

A schematic of the investigated waveguide is shown in Fig. 6a, where an infinitely long linear chain of Si cylindrical nanoparticles with a diameter *D*, a height *H* of 220 nm, and a gap of 50 nm $\left( P = D + G \right)$ are placed on top of a SiO2 substrate. Silicon material parameters are taken from Ref.^40^ while the SiO2 layer has been assumed non-dispersive in the spectral range of interest and modeled by a real refractive index of 1.45. The dominant transmission mode of this chain is the transverse magnetic (TM-like) mode. For the presented geometry, there are two similar TM modes, one for vertically oriented (parallel to the cylinder axis) and one for horizontally oriented (perpendicular to both the cylinder axis and the propagation direction) dipole modes of the cylindrical particles. The mode for the horizontal dipoles (along the *x*-axis) is the one to be investigated in this study. To that end, we excite the single unit cell of the structure with an infinitesimal *x*-directed magnetic dipole, i.e., with the magnetic current density $\vec M$ located on the central plane of the unit cell inside the Si nanoparticle, as shown in Fig. 6b. Taking the waveguide symmetry into account, we only analyze one-half of the structure after employing a perfect magnetic wall on the symmetry plane $x= 0$. This will save the computational resources and reduce the computation time significantly. For a given value of *N*, the normalized source frequency $\omega L/2\pi c$ is being evenly discretized. The synthesized FP configuration in this example corresponds to Fig. 9b discussed in “Methods”. We extract the complex *y*-component of the magnetic field, i.e., ${H_y}\left( {{{\vec {r}}_{obs}},\omega } \right)$, where ${\vec {r}_{obs}} = ({x_o},{y_o},z = 0)$ is a sample observation point with $({x_o},{y_o})$ is positioned inside the Si nanoparticle. Figure 7a represents the spectrum of the corresponding magnetic field intensity, i.e., $I\left( \omega \right) = {H_y}\left( {{{\vec {r}}_{obs}},\omega } \right) H_y^*\left( {{{\vec {r}}_{obs}},\omega } \right)$, when $N = 8$. Two resonant peaks are visible in the chosen wavelength range. To characterize the Bloch-resonance frequencies, based on Eq. (2), the EM fields on a longitudinal section of the resonator are computed. In this regard, the spatial distributions of ${\hat{z}}$ component of the electric field phasor at the above resonance frequencies are computed using Eq. (8) and shown in Fig. 7b,c. Final dispersion diagram of the desired TMx-polarized Bloch mode is retrieved With the same calculations for different values of *N* (Fig. 8a). In the same figure, the obtained results are compared with those we have calculated using the numerical FDTD band structure calculations^33^.

To evaluate the remaining modal parameters, the simulated spectral response has been fitted to the proposed model (Eq. 5) near the observed resonances. Firstly, the optimal initial guess value for the group velocity is explored by fitting the approximate dispersion relation of Eq. (9) to the obtained dispersion diagram (Fig. 8b). The unknown constants are computed as $c_0 = 12.77$, $c_1 = -144.7$, $c_2 = 676.3$, $c_3 = -1558$, $c_4 = -1765$, $c_5 = -778.9$. Thus, the attenuation constant and group velocity of the desired mode are straightforwardly characterized at resonance frequencies. The obtained results are compared with the results presented in Ref.^33^ (Fig. 8c). This diagram shows a reasonable agreement although there is a wavelength shift to longer wavelengths. This shift is rather expected and most probably originates from the difference between the material permittivities used in Ref.^33^ and those assumed in our simulations. The material parameters in Ref.^33^ were taken from ellipsometric measurements of the deposited films while the data reported by Ref.^40^ are adopted in this study. Figure 8d illustrates that the group velocities obtained using our proposed approach (blue circles), i.e., fitting Eq. (5) to the resonance line shapes observed in the frequency response of FP cavity, are well compared with those characterized using the approximate model of Eq. (9). The numerical results of this second example confirm, once again, that our proposed solution scheme based on a single unit-cell analysis is capable of characterizing complex modal properties of guided Bloch modes, even for 3D complicated geometries.

## Conclusion

Determining the propagation characteristics of guided Bloch modes is essential for exploiting periodic waveguides in nanophotonic devices. This paper first presents a comprehensive overview of existing techniques. Semi-analytical techniques require complex-root search, suffer from high computational costs, and are limited to simple geometries. Full-wave EM solvers yield phase constant but not accurate attenuation constant for open structures. The technique proposed in this research accurately calculates both the phase and attenuation constants from a real-valued function predicting optical intensity in a Fabry-Perot resonator. In other words, it combines the numerical results of commercial solvers with analytical post-processing for determination of complete dispersion diagrams. A single unit-cell technique is also presented to avoid high computational loads. For 2D and 3D radiative waveguides, our numerical results match literature values.

## Methods

### Derivation of the optical intensity spectrum

Let us assume that the FP resonator of Fig. 1b is an optical periodic waveguide terminated by two infinitely large PMC planes, as shown in Fig. 9a. As we have mentioned before, this FP resonator is composed of periodically repeated elements each symmetrical about the central plane of the unit cell. The resonator is excited by an electric current density located on the resonator midplane. When the excitation frequency is close to $\omega _l$ , i.e., the resonance frequency of the *l*-th mode of the FP resonator, the EM field inside the FP resonator is dominated by the corresponding mode field of the optical periodic waveguide. Hence, the total EM field can be viewed as a superposition of multiple forward and backward traveling waves each of which governed by that particular eigen-mode of the optical periodic waveguide which is dominant at $\omega = \omega _l$. We assume that this eigen-mode is the *m*-th mode of the optical periodic waveguide which has a complex propagation coefficient given by ${\gamma _m}\left( \omega \right) = {\alpha _m}\left( \omega \right) + j{\beta _m}\left( \omega \right)$. Under the above assumption, the infinite series of forward-traveling waves begins with $\vec {E}_{m,0}^ + \left( {{{\vec {r}}_{obs,1}}} \right)$ as the complex amplitude of the *m*-th eigen-mode of the optical periodic waveguide. It is measured on the right-hand side of $z = 0$ plane at the observation point ${\vec {r}_{obs,1}} = \left( {{x_o},{y_o},{z_o} = {0^ + }} \right)$ , and travels to the right (designated by the superscript “+”). The complex amplitude of the succeeding forward-traveling wave, i.e., after a complete round-trip, can be formulated as

10 $$\begin{aligned} \vec {E}_{m,1}^ + \left( {{{\vec {r}}_{obs,1}}} \right) = \Gamma _{\mathrm{{PMC}}}^2\exp \left( { - 2{\gamma _m}L} \right) \vec {E}_{m,0}^ + \left( {{{\vec {r}}_{obs,1}}} \right) \end{aligned}$$

where $\Gamma _{PMC} = 1$ is the reflection coefficient of the PMC mirrors, and *L* is the resonator length ($L = NP$ for periodic waveguides). Following the same procedure, the complex amplitudes of the next forward-traveling waves can be determined. The total forward-traveling electric field of the *m*-th eigenmode at the position of ${\vec {r}_{obs,1}}$ is denoted by $\vec {E}_m^ + \left( {{{\vec {r}}_{obs,1}}} \right)$ and can be calculated as the sum of an infinite geometrical series. The result of this sum is

11 $$\begin{aligned} \vec {E}_m^ + \left( {{{\vec {r}}_{obs,1}}} \right) = \frac{1}{{1 - \Gamma _{\mathrm{{PMC}}}^2\exp \left( { - 2{\gamma _m}L} \right) }}\vec {E}_{m,0}^ + \left( {{{\vec {r}}_{obs,1}}} \right) \end{aligned}$$

The total field returning from the PMC mirror on the right-hand side and traveling to the left (indicated in Fig. 9a by the superscript “-”) is obviously given by

12 $$\begin{aligned} \vec {E}_m^ - \left( {{{\vec {r}}_{obs,1}}} \right) = {\Gamma _{\mathrm{{PMC}}}}\exp \left( { - {\gamma _m}L} \right) \vec {E}_m^ + \left( {{{\vec {r}}_{obs,1}}} \right) \end{aligned}$$

at $\vec {r}_{obs}$. Therefore, the total electric field of the *m*-th eigenmode on the right-hand side of $z = 0$, i.e., at $\vec {r}_{obs,1}$ is obtained as

13 $$\begin{aligned} {{\vec {E}}_m}\left( {{{\vec {r}}_{obs,1}}} \right)&= \vec {E}_m^ + \left( {{{\vec {r}}_{obs,1}}} \right) + \vec {E}_m^ - \left( {{{\vec {r}}_{obs,1}}} \right) \nonumber \\&= \frac{1}{{1 - {\Gamma _{\mathrm{{PMC}}}}\exp \left( { - {\gamma _m}L} \right) }}\vec {E}_{m,0}^ + \left( {{{\vec {r}}_{obs,1}}} \right) \end{aligned}$$

Similarly, the total electric field of the *m*-th eigenmode on the left-hand side, i.e., at ${\vec {r}_{obs,2}} = \left( {{x_o},{y_o},{z_o} = {0^ - }} \right)$

14 $$\begin{aligned} {\vec {E}_m}\left( {{{\vec {r}}_{obs,2}}} \right) = \frac{1}{{1 - {\Gamma _{\mathrm{{PMC}}}}\exp \left( { - {\gamma _m}L} \right) }}\vec {E}_{m,0}^ - \left( {{{\vec {r}}_{obs,2}}} \right) \end{aligned}$$

Accordingly, the total electric field of the *m*-th eigenmode at an observation point on the resonator midplane, i.e., at ${\vec {r}_{obs}} = \left( {{x_o},{y_o},{z_o} = 0} \right)$ is given by ${\vec {E}_m}\left( {{{\vec {r}}_{obs}}} \right) = {{\left[ {{{\vec {E}}_m}\left( {{{\vec {r}}_{obs,1}}} \right) + {{\vec {E}}_m}\left( {{{\vec {r}}_{obs,2}}} \right) } \right] } \big / 2}$. The initial electric fields on the right-hand side of the plane $z = 0$ can be decomposed in the transverse and longitudinal components, i.e.,

15 $$\begin{aligned} \vec {E}_{m,0}^ + \left( {{{\vec {r}}_{obs,2}}} \right) = \vec {E}_{u,m,0}^ + \left( {{{\vec {r}}_{obs,1}}} \right) - {{\hat{z}}}E_{z,m,0}^ + \left( {{{\vec {r}}_{obs,1}}} \right) \end{aligned}$$

where *u* being a linear combination of ${\hat{x}}$ and ${\hat{y}}$ represents the transverse components of the electric field. Due to the existing symmetry about the resonator midplane, one can obtain the total electric field on the midplane as

16 $$\begin{aligned} {\vec {E}_m}\left( {{{\vec {r}}_{obs}}} \right) = \frac{1}{{1 - {\Gamma _{\mathrm{{PMC}}}}\exp \left( { - {\gamma _m}L} \right) }}\vec {E}_{u,m,0}^ + \left( {{{\vec {r}}_{obs,1}}} \right) \end{aligned}$$

As a consequence, the electric field intensity at a sample observation point on the midplane is obtained using the relation ${\left| {{{\vec {E}}_m}\left( {{{\vec {r}}_{obs}}} \right) } \right| ^2} = {\vec {E}_m}\left( {{{\vec {r}}_{obs}}} \right) \cdot \vec {E}_m^*\left( {{{\vec {r}}_{obs}}} \right)$ as

17 $$\begin{aligned} {I_m}\left( \omega \right) = \frac{{{C_m}{e^{ + {\alpha _m}\left( \omega \right) L}}}}{{\cosh \left( {{\alpha _m}\left( \omega \right) L} \right) - {\Gamma _{\mathrm{{PMC}}}}\cos \left( {{\beta _m}\left( \omega \right) L} \right) }} \end{aligned}$$

where $C_m$ is a real-valued constant.

In the dual case, we assume the FP resonator under investigation is terminated by two PEC planes and is excited by a magnetic current density located on the resonator midplane (as shown in Fig. 9 (b)). Here, $\vec {H}_{m,0}^ + \left( {{{\vec {r}}_{obs,1}}} \right)$ plays the role of $\vec {E}_{m,0}^ + \left( \vec {r}_{obs,1}\right)$; therefore, the dual relation of Eq. (10) can be formulated as

18 $$\begin{aligned} \vec {H}_{m,1}^ + \left( {{{\vec {r}}_{obs,1}}} \right) = {\left( {\Gamma _{PEC}^H} \right) ^2}\exp \left( { - 2{\gamma _m}L} \right) \vec {H}_{m,0}^ + \left( {{{\vec {r}}_{obs,1}}} \right) \end{aligned}$$

and the dual relation of Eq. (11) is

19 $$\begin{aligned} \vec {H}_m^ + \left( {{{\vec {r}}_{obs,1}}} \right) = \frac{1}{{1 - {{\left( {\Gamma _{PEC}^H} \right) }^2}\exp \left( { - 2{\gamma _m}L} \right) }}\vec {H}_{m,0}^ + \left( {{{\vec {r}}_{obs,1}}} \right) \end{aligned}$$

Again, the dual relation of Eq. (12) is given by

20 $$\begin{aligned} \vec {H}_m^ - \left( {{{\vec {r}}_{obs,1}}} \right) = \Gamma _{PEC}^H\exp \left( { - {\gamma _m}L} \right) \vec {H}_m^ + \left( {{{\vec {r}}_{obs,1}}} \right) \end{aligned}$$

Therefore, the total magnetic field of the *m*-th eigenmode on the right-hand side of $z = 0$ at $\vec {r}_{obs,1}$ is obtained as

21 $$\begin{aligned} {{\vec {H}}_m}\left( {{{\vec {r}}_{obs,1}}} \right)&= \vec {H}_m^ + \left( {{{\vec {r}}_{obs,1}}} \right) + \vec {H}_m^ - \left( {{{\vec {r}}_{obs,1}}} \right) \nonumber \\&= \frac{1}{{1 - \Gamma _{PEC}^H\exp \left( { - {\gamma _m}L} \right) }}\vec {H}_{m,0}^ + \left( {{{\vec {r}}_{obs,1}}} \right) \end{aligned}$$

Similarly, the total magnetic field of mode *m* on the left side at ${\vec {r}_{obs,2}} = \left( {{x_o},{y_o},{z_o} = {0^ - }} \right)$ can be written as

22 $$\begin{aligned} {\vec {H}_m}\left( {{{\vec {r}}_{obs,2}}} \right) = \frac{1}{{1 - \Gamma _{PEC}^H\exp \left( { - {\gamma _m}L} \right) }}\vec {H}_{m,0}^ - \left( {{{\vec {r}}_{obs,2}}} \right) \end{aligned}$$

The dual relation of Eq. (15) is

23 $$\begin{aligned} \vec {H}_{m,0}^ + \left( {{{\vec {r}}_{obs,2}}} \right) = \vec {H}_{u,m,0}^ + \left( {{{\vec {r}}_{obs,1}}} \right) - {{\hat{z}}}H_{z,m,0}^ + \left( {{{\vec {r}}_{obs,1}}} \right) \mathrm{{,}} \end{aligned}$$

which results in

24 $$\begin{aligned} {\vec {H}_m}\left( {{{\vec {r}}_{obs}}} \right) = \frac{1}{{1 - \Gamma _{PEC}^H\exp \left( { - {\gamma _m}L} \right) }}\vec {H}_{u,m,0}^ + \left( {{{\vec {r}}_{obs,1}}} \right) \mathrm{{.}} \end{aligned}$$

Consequently, the magnetic field intensity at a sample observation point on the midplane $\vec {r}_{obs}$ is obtained using the relation ${\left| {{{\vec {H}}_m}\left( {{{\vec {r}}_{obs}}} \right) } \right| ^2} = {\vec {H}_m}\left( {{{\vec {r}}_{obs}}} \right) \cdot \vec {H}_m^*\left( {{{\vec {r}}_{obs}}} \right)$ as

25 $$\begin{aligned} {I_m}\left( \omega \right) = \frac{{{D_m}{e^{ + {\alpha _m}\left( \omega \right) L}}}}{{\cosh \left( {{\alpha _m}\left( \omega \right) L} \right) - \Gamma _{PEC}^H\cos \left( {{\beta _m}\left( \omega \right) L} \right) }} \end{aligned}$$

where $D_m$ is a real-valued constant.

It should be noted that the reflection coefficient for the tangential magnetic field $\Gamma _{PEC}^H$ satisfies $\Gamma _{PEC}^H =-\Gamma _{PEC}$ where$\Gamma _{PEC}$ refers to the reflection coefficient for the tangential electric field.

### Calculation of the group velocity

As explained previously, the attenuation constant and group velocity of the Bloch modes are evaluated by fitting Eq. (5) to the optical intensity spectrum obtained by means of a full-wave simulation. For instance, the nonlinear optimization toolbox available in MatLab can be utilized for this purpose. However, to carry out a nonlinear fitting process, one requires the initial guess values as close as possible to the actual value in order to achieve fast convergence with the desired numerical accuracy. Here, there is the possibility of finding a proper initial guess for the group velocity $v_g$ by using the calculated dispersion diagram. To this end, we model the dependence of the propagation phase constant on the frequency $\omega$ by

26 $$\begin{aligned} \frac{1}{{k_0}P} = f\left( {\beta P} \right) = \sum \limits _{n = 0}^\infty {\frac{{{c_n}}}{{{{\left( {\beta P} \right) }^n}}}} \end{aligned}$$

where $k_0 = \omega /c$ is the wavenumber in vacuum and *c* is the speed of light. We readily fit the above analytical function to our computationally calculated dispersion diagram. The group velocity as a function of frequency can thus be approximated directly using the relation ${v_g}\left( \omega \right) = {{d\omega } / {d\beta }}$. This leads to a highly accurate value for $v_g$ at resonance frequencies, and simplifies the curve fitting procedure for obtaining the attenuation constant. This leads to a highly accurate value for $v_g$ at resonance frequencies and simplifies the curve-fitting procedure for obtaining the attenuation constant.

## Data Availability

The datasets used and analyzed during the current study are available from the corresponding author upon reasonable request.
